# Comparative life cycle assessment of a novel sustainable road pavement system adopting recycled plastic from PET bottles and carbonated aggregate

**DOI:** 10.1016/j.heliyon.2024.e24354

**Published:** 2024-01-09

**Authors:** Ottavia Rispoli, Oluwatoyin Opeyemi Ajibade

**Affiliations:** Civil and Building Services Engineering Division, School of The Built Environment and Architecture, London South Bank University, 103 Borough Road, London, SE1 0AA, UK

**Keywords:** Asphalt, Carbonated aggregate, Life cycle impact assessment, Sustainable road pavement system, Waste plastic recycling

## Abstract

Road surfacing is crucial in improving community accessibility and mobility. Adopting sustainable measures is extremely important to prevent the significantly high environmental burdens associated with road pavement systems production. This study presents a comparative life cycle assessment of traditional pavement systems and their sustainable alternatives made and tested at the London South Bank University laboratories. The low-impact pavement mixes proposed herein provide a novel, innovative method for producing sustainable road systems. Unlike traditional approaches, these asphalt mixes utilise materials derived from recycled polyethylene terephthalate (plastics) and carbonated aggregates and are produced at temperatures significantly lower (warm mix).

The Simapro software (Consultants, 2023) [1] was used to model the analysed asphalt mixes, and all life cycle inputs and outputs were characterised during the life cycle impact assessment phase into potential impacts using the IMPACT World + Midpoint method. Additionally, an uncertainty analysis employing Monte Carlo simulation was conducted to validate the life cycle assessment findings, reinforcing the robustness and credibility of this study's results. Notably, the assessment shows substantial reductions in the environmental impact of road pavement mixes adopting recycled plastic and carbonated aggregates, with various outcomes. Compared to traditional mixes, Climate Change emissions are reduced by approximately 40 %–60 %, Marine Eutrophication exhibits reductions of up to 30 %, and Freshwater Eutrophication decreases by up to 20 %. These findings suggest that integrating this sustainable road pavement approach can significantly reduce the environmental burdens of asphalt production and give asphalt a pivotal role in tackling waste reduction, carbon sequestration, and achieving Net Zero. Also, the proposed system can positively contribute to the current United Kingdom's (UK) circular policy model by reconsidering current waste management frameworks and integrating more efficient settings.

## Abbreviations

CA: Carbonated Aggregate
LCA: Life Cycle Assessment
LCIA: Life Cycle Impact Assessment
LSBU: London South Bank University
PET: Polyethylene Terephthalate (Plastic)
RPS: Road Pavement Systems
SRPS: Sustainable Road Pavement Systems
WM: Warm Mixes
HM: Hot Mixes

## 1 Introduction

With the increase in construction of paving asphalt driven by aging infrastructure rehabilitation and increasing urbanisation and industrialisation in developing regions, the global asphalt market size was estimated at 119.4 million tonnes in 2020 and is projected to reach 183 million tonnes [1] by 2027 [2,3]. In Great Britain alone, 95 % of the 247,500 miles of road require resurfacing at the end of their design life, and it is estimated that over 25 million tonnes of asphalt are produced every year in the UK [4], partially to respond to the demand. Nonetheless, the quarrying operations linked to procuring the materials traditionally used in asphalt mixes cause substantial environmental damage, including modifications of the hydrologic system, habitat alterations, and soil loss [5]. Similarly, a considerable proportion of the global embodied carbon emissions arises from the manufacturing procedures of materials used in road pavement systems [6]. The processes include dredging virgin gravel and sand and their transportation to the point of use. Therefore, transitioning into a circular economy necessitates the development of novel sustainable materials when producing environmentally friendly solutions for road pavement systems [7]. Carbon dioxide capture, utilisation, and storage are actions that are deemed necessary by the Intergovernmental Panel on Climate Change and the International Energy Agency to achieve a net-zero future [8]. Incorporating Carbonated Aggregate (CA) into the Sustainable Road Pavement System (SRPS) presented in this paper addresses the pressing need for technologies that utilise CO_2_ for beneficial applications. It also demonstrates the potential for integrating its adoption within the wider infrastructure market, influencing global emissions on a long-term basis. CAs are made in commercial quantities in the UK and other parts of the world using methods that include Air Pollution Control residue (APCr) [[9], [10], [11]]. This technology finds application in making the CA adopted in the SRPS [10,12]. Likewise, polymers such as waste plastics (e.g. polyethylene terephthalate (PET) plastics included in the SRPS) replace a proportion of bitumen content. Utilising recycled PET aligns with the global call for industries to transition to a circular economy model [13,14]. At the same time, similarly to CA, by adopting secondary materials from plastic bottles natural resources depletion is reduced. Furthermore, this is a beneficial reuse alternative for 1.3 billion tonnes of waste plastics that are estimated to end up in landfills globally by 2040 because the data provided by the British Plastic Federation shows that only 45 % of UK-generated plastic wastes are recycled [15].

Repurposing the waste plastics and incorporating carbonated aggregates into asphalt present innovative strategies to regain precious land areas currently dedicated to landfill use, consuming a large amount of CO_2_ from heavy industries, and protecting decreasing reserves of natural aggregate (NA) (e.g. sand and gravel), and the environmental impacts of dredging and quarrying.

The proposed sustainable pavement's novelty lies in the adoption of Warm Mix (WM) methods, which aligns with Highways England's call for accelerating its adoption as a standard across the supply chain of road pavement construction [16]. The WM (mixing at a lower temperature) is applied to further improve the sustainable mix environmental performance. To achieve the intended consistency and properties of the asphalt mixture, conventional asphalt mix production requires heating the binder and aggregates to temperatures within the range of 170 °C–180 °C. However, the SRPS mixes were made using lower temperatures (ranging from 145 °C to 155 °C), consequently achieving energy savings.

In this context, to meet the United Kingdom's (UK) target of achieving net zero emissions by 2050 and establish a sustainable strategy that protects against the disastrous outcomes of global warming, new technologies for asphalt production (WM) and secondary materials derived from PET recycling or CA are adopted in the sustainable pavement mixes presented in this study. Previous research has looked into utilising plastic as a coating for recycled aggregate [17] or recycled aggregate by carbonation treatment in combination with WM [18], however, none of the studies have looked into combining these three sustainable approaches into one unique technology as this research proposes.

## 2 Materials and methodology

### 2.1 SRPS mix

**Fig. 1:**
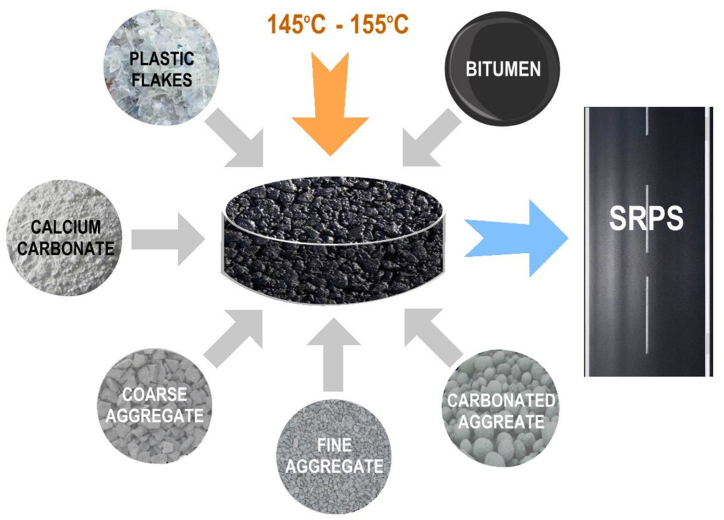
Sustainable Road Pavement System mixing process.

To enable the Life Cycle Assessment (LCA) of the SRPS, a set of samples was made (Table 1; Fig. 1) using the MS-2 Marshall Analysis Method [19].

**Table 1:** Control sample and Sustainable Road Pavement System mix description.

Mix No.	Mix Ref.	Mix Description
**1**	**CS**	Control sample
**2**	**10P-0CA**	10 % of the bitumen content of the control sample is replaced with waste PET bottles.
**3**	**15P-0CA**	15 % of the bitumen content of the control sample is replaced with waste PET bottles.
**4**	**0P-15CA**	15 % of the coarse natural aggregates (10 % of the total aggregates in the control sample) were replaced with carbonated manufactured aggregates.
**5**	**0P-37CA**	37 % of the fine natural aggregates (10 % of the total aggregates in the control sample) were replaced with carbonated manufactured aggregates.
**6**	**10P-15CA**	10 % of the bitumen content of A is replaced with waste PET bottles, and 15 % of the coarse natural aggregates (10 % of the total aggregate in the control sample) are replaced with carbonated manufactured aggregates.
**7**	**10P-27CA**	27 % of the total natural aggregate in the control sample is replaced with carbonated aggregate, and 10 % of the BC is replaced with waste PET. BC was 4.5 %, 5.0 %, 5.5 %, and 6.0 %
**8**	**10P-50CA**	50 % of the total natural aggregate in the control sample is replaced with carbonated aggregate, and 10 % of BC is replaced with PET.

The Marshall Analysis involved the determination of the proportion of natural aggregates (nominal maximum particle size 12.5 mm), optimum bitumen content (5 %), and proportion of milled limestone powder as filler (6 %) (see Table 1). Likewise, the proportion of carbonated aggregate (<5 mm particle sizes) and shredded waste PET (<5 mm) were determined (see Table 1). Each asphalt sample was compacted with 75 blows on each side using the Marshall hammer and the Marshall stability and flow were determined. The design air void for the SRPS mixes was obtained as 4 %, voids in mineral aggregate >14 %, and voids filled with asphalt between 65 and 75 %.

As presented in Table 1, the naming convention adopted for each Sustainable RPS sample relates to their specific composition or mixture. For example, sample "10P-0CA" represents a specific composition or mixture, where "10P″ indicates a sample with 10 % plastic content and "0CA" indicates no carbonated aggregate adopted in the mix (0 %). The mixes contain variable content of.

### 2.2 Goal and scope

The LCA aims to evaluate the environmental impact of seven innovative SRPS mixes (items 2 to 8, Table 1) and compare it to the control sample (item 1, Table 1) and each innovative mix. The approach adopted in this study to design the various LCA phases, including the inventory and impact analysis, is documented in the ISO 14040 and ISO 14044 standards [20,21].

**Fig. 2:**
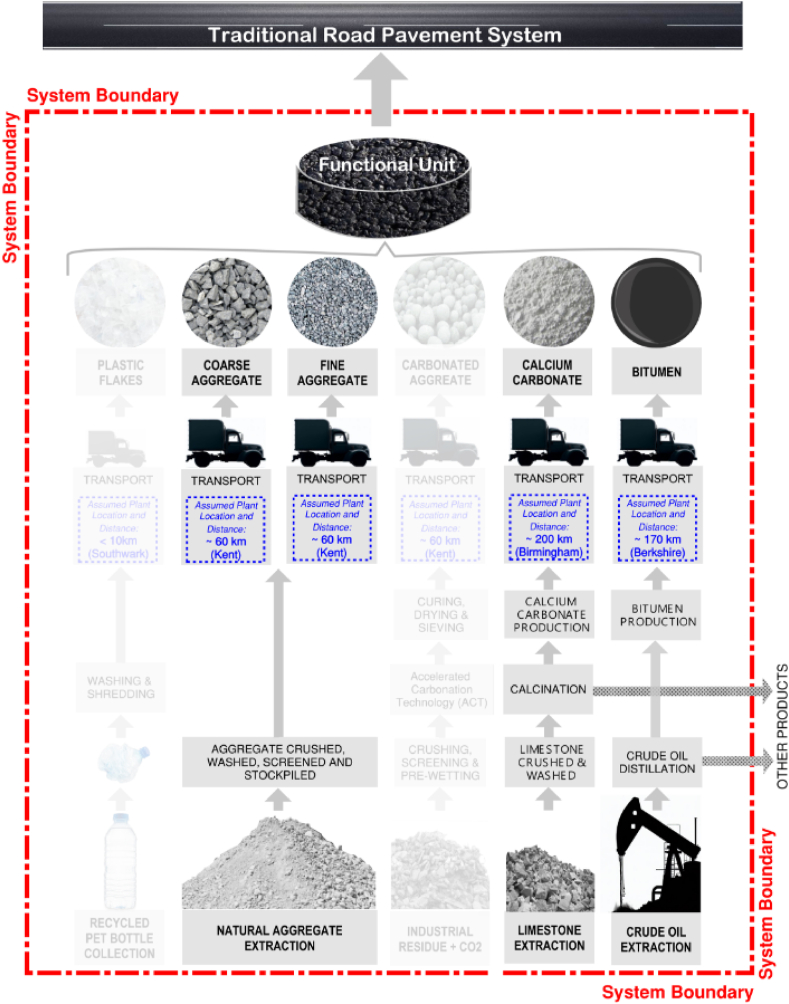
LCA system boundary for Road Pavement System mix.

A cradle-to-gate approach was adopted, including all the processes involved in sourcing and manufacturing the materials constituting the RPS (Fig. 2) but excluding the resulting pavement's usage, construction and maintenance phase. Since this research involves comparing different asphalt mixes that exhibit similar performance levels but are composed of different materials, omitting these stages is deemed suitable since all pavements, irrespective of the asphalt mixes employed, would undergo identical construction methods, maintenance regime, and service life [[22], [23], [24], [25]]. Similarly, this comparative analysis neglects the pavement's end-of-life phase. It assumes that after blending various materials to form each asphalt mix and using them for new pavement construction, they would all undergo identical disposal treatment.

### 2.3 Functional unit

The functional unit (FU) defines the road mixes CS to 10P-50CA (Table 1) and provides a valuable reference for scaling the input and output data in every stage of the LCA [22]. Using a functional unit representative of the road mixes also provides a reference by which LCA results are normalised, and data can be expressed on a common basis in the comparison study [26].

**Fig. 3:**
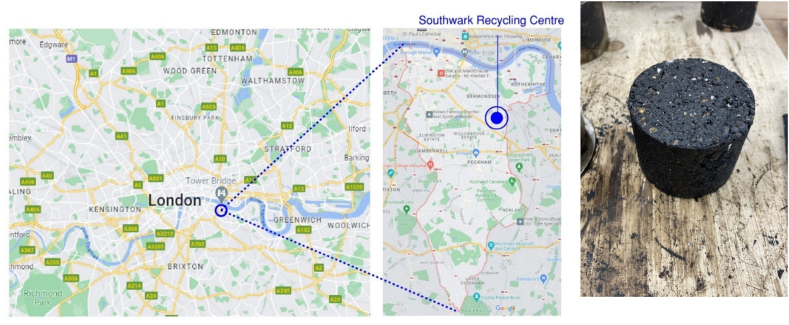
Illustration of the waste plastic recycling location and a sample of asphalt produced.

This research represents the functional unit using a 1 km asphalt mix produced in the London Borough of Southwark, United Kingdom (Fig. 3). The benefit of specifying the expected use and location of the functional unit lies in the opportunity to conduct further evaluations concerning transportation, material manufacturing, and resource acquisition. The study focuses on a road width of 9 m, which is representative of the standard dimensions found in Southwark.

Another factor typically considered when defining the functional unit is its intended design life which, for asphalt, would be, on average, 10–15 years. However, for this study, the assumption is that the lifespan of the asphalt mix design remains unaffected by the choice of mix used in road resurfacing, thus making it unnecessary for the functional unit's characterisation [22].

### 2.4 System boundary

When defining the System Boundary (SB) for the recycled materials constituting the SRPS and RPS mixes, the burdens associated with producing the primary materials required for obtaining secondary materials, such as recycled plastic for PET bottles, are excluded. This is in line with the SB Cut-Off approach to recycling. This assumption was based on the idea that recycled materials would only be accountable for primary production processes after the secondary life starts [27].

### 2.5 Life cycle inventory (LCI)

#### 2.5.1 General

Three primary data sources are adopted: surveys, literature and the Ecoinvent database, version 3.6, directly accessible through the Simapro software used for the analysis (Table 2). The study uses the Ecoinvent database to determine material production, such as quarrying and transport to plant activities or material processing from virgin materials. Literature is referred to whenever the Ecoinvent database does not provide exhaustive information. In the mixture production phase, for instance, the energy needed to blend the asphalt components and increase their temperatures to the desired levels, whether using HM or WM, is drawn from the research conducted by Ma et al. [28]. Similarly, for bitumen production, a mass allocation is considered based on the assumption that 4.48t of crude oil is required to produce 1t bitumen [29] (see Table 3).

**Table 2:** – Life Cycle Assessment Data sources per process.

LCA Phase	Material/Product	Process	Source
**Material specification**	Asphalt design	Mass quantities	From the Marshall Analysis
**Material sourcing**	Bitumen	Straight-run refinery production from crude oil	Eurobitume (2011) and Blomberg et al. (2012)
	Bitumen	Mass allocation from crude oil	Eurobitume (2011) and Blomberg et al. (2012)
	Coarse/Fine Aggregate	Natural Aggregate (NA) quarrying	*“Gravel, round gravel and sand quarry operation”* – Ecoinvent database survey data
	Carbonated Aggregate (CA)	CA sourcing from primary materials	VICAT-Carbon8 (2023)
	PET plastic	PET plastics transport to the recycling plant	*“Municipal waste collection service by 21 metric ton lorry”* – Ecoinvent database survey data
	Filler	Calcium carbonate quarrying operations	*“Limestone quarry operation.”* – Ecoinvent database survey data
**Material processing**	Bitumen	Seal production and transport to the plant	*“Bitumen seal production”* – Ecoinvent database survey data
	Coarse/Fine Aggregate	NA processing and crushing	Included in: *“Gravel, round gravel and sand quarry operation”* – Ecoinvent database survey data
	Carbonated Aggregate	CA processing	VICAT-Carbon8 (2023)
	Carbonated Aggregate, and Coarse/Fine Aggregate	Transport to mixing plants	*“Transport, freight, lorry 16–32 metric ton, euro6”* – Ecoinvent database survey data
	Filler and Bitumen	Transport to mixing plants	*“Transport, freight, lorry 36 metric ton, euro6”* – Ecoinvent database survey data
	PET plastic	Transport to mixing plants	*“Municipal vehicle”* – Ecoinvent database survey data
	PET plastic	PET plastics processing	*Plastic flake for recycling*
	PET plastic	PET Transport to mixing plants	*“Municipal waste collection service by 21 metric ton lorry”* – Ecoinvent database survey data
	Filler	Calcium carbonate processing	*“Calcium carbonate”* – Ecoinvent database survey data
	Warm Asphalt Mix (WAM)	Mixing processes, including heating up to 180 °C	Ma et al. (2019)
	Hot Asphalt Mix (HAM)	Mixing processes, including heating up to 145 °C	Ma et al. (2019)

**Table 3:** – LCA results per asphalt mix considered in the study (Note: eq represents equivalent).

Impact category	Unit	Mixes Reference							
		CS	10P-0CA	15P-0CA	0P-15CA	0P-37CA	10P-15CA	10P-27CA	10P-50CA
**Climate change, short term**	kg CO_2_ eq	1.74E+05	1.36E+05	1.36E+05	1.31E+05	1.31E+05	1.26E+05	1.02E+05	7.91E+04
**Climate change, long term**	kg CO_2_ eq	1.66E+05	1.28E+05	1.28E+05	1.23E+05	1.23E+05	1.18E+05	9.53E+04	7.20E+04
**Photochemical oxidant formation**	kg NMVOC eq	6.72E+02	5.07E+02	5.07E+02	5.25E+02	5.25E+02	5.05E+02	4.78E+02	4.73E+02
**Ozone layer depletion**	kg CFC-11 eq	5.38E-02	4.76E-02	4.76E-02	5.09E-02	5.09E-02	4.76E-02	4.46E-02	4.46E-02
**Human toxicity cancer**	CTUh	9.39E-03	8.94E-03	8.94E-03	9.22E-03	9.22E-03	8.88E-03	8.39E-03	8.25E-03
**Human toxicity non-cancer**	CTUh	1.75E-02	1.70E-02	1.70E-02	1.72E-02	1.72E-02	1.70E-02	1.61E-02	1.61E-02
**Terrestrial acidification**	kg SO_2_ eq	1.63E+00	1.21E+00	1.21E+00	1.26E+00	1.26E+00	1.20E+00	1.14E+00	1.13E+00
**Freshwater eutrophication**	kg PO_4_ eq	8.12E+00	7.15E+00	7.15E+00	7.66E+00	7.66E+00	7.14E+00	6.65E+00	6.63E+00
**Marine eutrophication**	kg N eq	1.15E+01	8.43E+00	8.43E+00	8.68E+00	8.68E+00	8.38E+00	7.95E+00	7.84E+00

#### 2.5.2 Transport

**Fig. 4:**
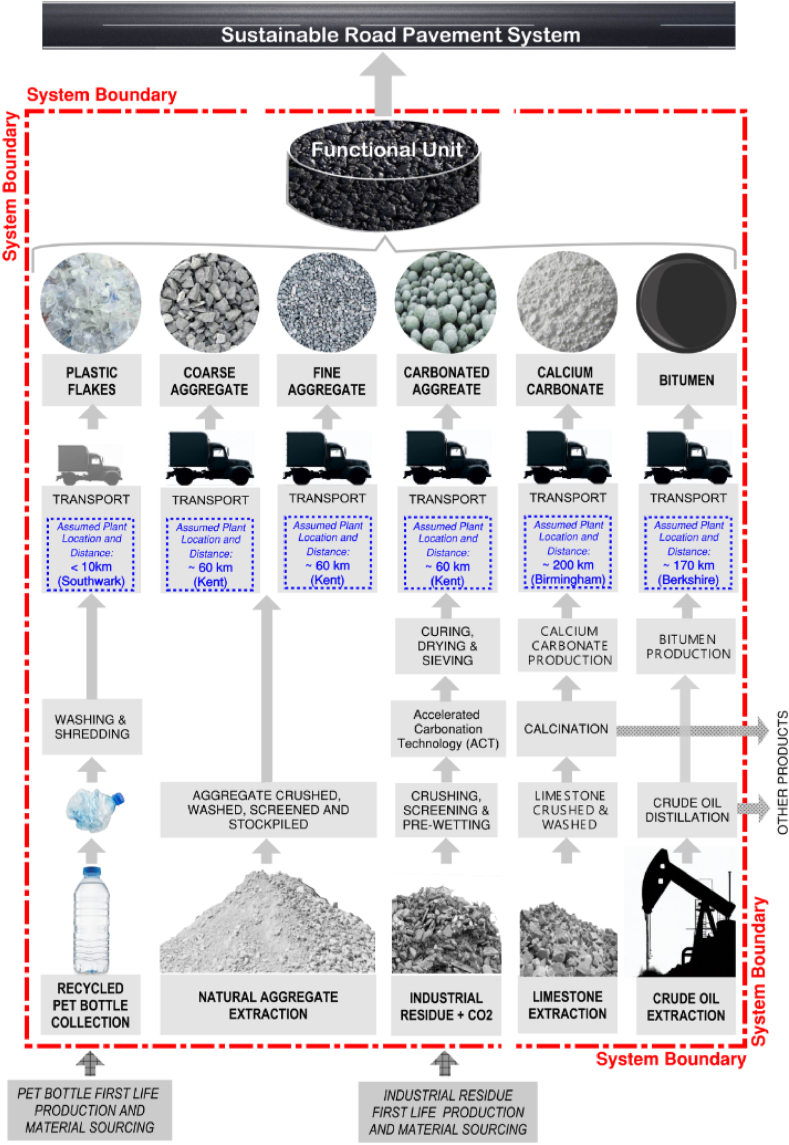
Life Cycle Assessment system boundary and processes for Sustainable Road Pavement System.

A default distance of 60 km between the quarries and the mixing plants is assumed for the fine and coarse aggregate, while 200 km and 170 km, are used for calcium carbonate and bitumen, respectively. Fig. 3, Fig. 4 indicate the assumed plant locations.

60 km was also assumed for the CA as the VICAT-Carbon8's plants closest to the London Borough of Southwark site are in Medway, Kent (Fig. 4).

To account for the distance between the Southwark household reuse and recycling centre and the farthest point within the borough boundary, the distance for recycled PET plastic is reduced to 10 km. (Fig. 4).

#### 2.5.3 Allocation

The environmental impacts of asphalt mix CS to 10P-50CA were evaluated using the Ecoinvent version 3 cut-off model. The cut-off model complies with an attributional interpretation of environmental impact assessments [26,30,31], focusing on assessing what part of the global environmental burdens belongs to the asphalt mixes under consideration [32,33]. Furthermore, the European Product Environmental Environmental Footprint guideline recommends the attributional model approach [[34], [35], [36]].

The analysis utilised a mass allocation method for the output, allocating co-products based on their mass, and determining their environmental impacts. While ISO 14044 (**2006a**) suggests avoiding allocation whenever possible, it becomes necessary in this study due to integrating various materials within a single asphalt mix. However, a mass allocation approach was deemed suitable, as it establishes a physical relationship between each of the asphalt constituents, avoids neglecting their environmental impact, and allows the product outputs partitioning [20,21,37].

When defining the allocation method of carbonated aggregate (CA), the available data for the product in the VCAT-Carbon8 documentation [12] is used and modelled within the Simapro analysis. Unlike the PET recycling processes, the CA has no co-products to be allocated. However, the energy consumption and hazardous waste amounts were allocated as per VICAT-Carbon8 data [12]. A 1 % cut-off is applied with the elementary flows to and from the CA, contributing to a minimum of 99 % of the declared environmental impacts accounted for in the analysis [12].

### 2.6 Life cycle impact assessment (LCIA)

The SimaPro software, version 9.1.1 [38], developed by PRé Consultants in the Netherlands, was used to model processes, life cycle inputs, and outputs related to the functional unit under analysis. The Life Cycle Impact Assessment (LCIA) calculation methodology employed in this study is IMPACT World + Midpoint v 1.01 [1], created collaboratively by RIVM, Radboud University, Norwegian University of Science and Technology, and PRé Sustainability. To incorporate the CA data, only the impacts included in the VCAT-Carbon8 document [12] have been considered, and these include Climate Change short-term [CCST], Climate change long-term [CCLT], Photochemical oxidant formation [POF], Ozone layer depletion [OLD], Human toxicity cancer [HTC], Human toxicity non-cancer [HTNC], Terrestrial acidification [TA], Freshwater eutrophication [FE], Marine eutrophication [ME].

## 3 Results and discussion

### 3.1 Comparison of the environmental impacts of all mixes

Table 3 summarises the Life Cycle Assessment (LCA) results for all asphalt mixes calculated per functional unit.

**Fig. 5:**
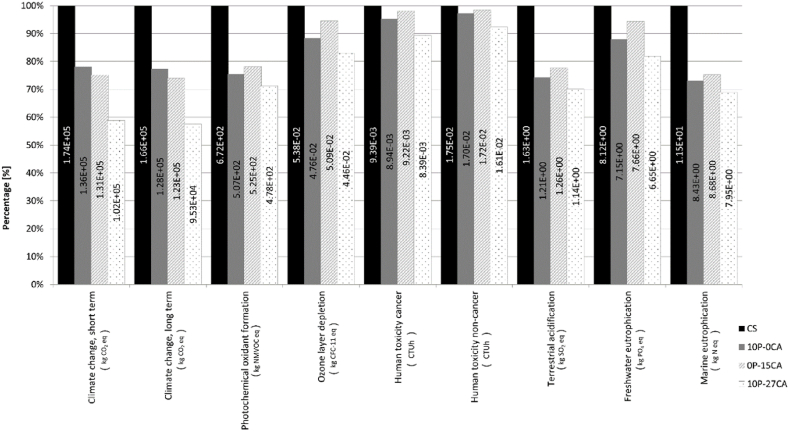
– IMPACT World + results (Midpoint method), Sustainable Road Pavement System mixes compared to Road Pavement System Control Sample.

Fig. 5 demonstrates the variations in impact categories among various mixes, with greater gap values indicating more substantial enhancements in environmental efficiency. Noteworthy improvements are observed in the mix that solely adopts plastic (10P-0CA), showing significant enhancements in all impact categories. A noteworthy 20–25 % improvement is observed in Terrestrial Acidification, Climate Change, Photochemical Oxidant Formation, and Marine Eutrophication.

**Fig. 6:**
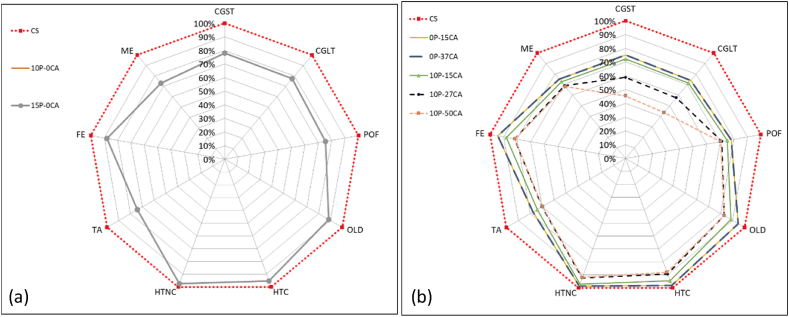
– (a) Variation of impact categories variations across Sustainable Road Pavement System mix 10P-0CA and 15P-0CA compared to the Control Sample. (b) Variation of impact categories variations across Sustainable Road Pavement System mix 0P-15CA, 0P-37CA, 10P-15CA, 10P-27CA and 10P-50CA compared to the control sample CS.

**Fig. 7:**
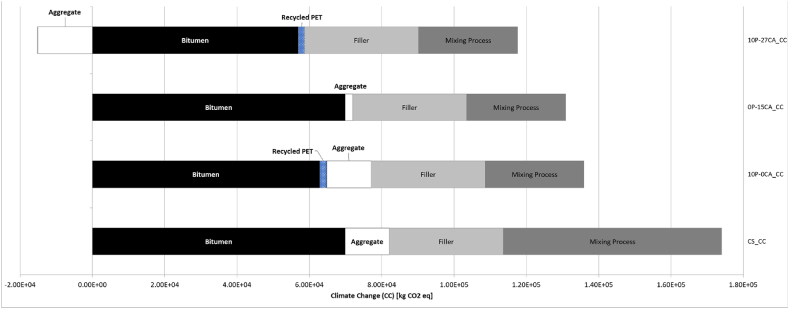
– LCA results for Climate change associated with individual materials and operations.

**Fig. 8:**
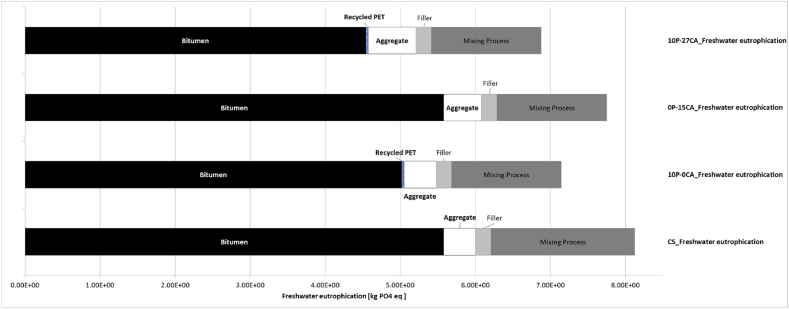
– LCA results for Freshwater eutrophication associated with individual materials and operations.

**Fig. 9:**
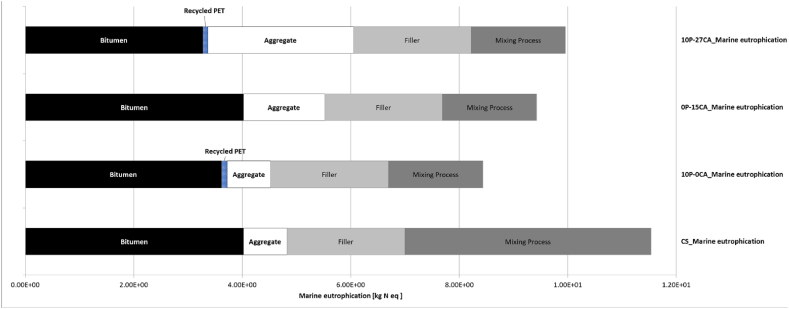
– LCA results for Marine Eutrophication associated with individual materials and operations.

Based on the analysis provided in Fig. 7, this decrease in emissions, when comparing the results to the control sample, is associated with a 5tCO2eq/FU reduction in bitumen-associated Climate Change emissions, which are replaced with lower carbon processes such as those associated with recycled plastic processes. Upon analysing the results, however, the same behaviour is not displayed in the other categories where bitumen replacement with recycled PET has minimal effect, as shown for Freshwater Eutrophication and Marine Eutrophication (Fig. 8, Fig. 9). As evident for mix 10P-0CA, the adoption of warm mixing methods is primarily responsible for its reduced environmental impact. This is demonstrated in Fig. 7, Fig. 9, showing an apparent decrease of more than 50 % in emissions associated with the SRPS mixing processes compared to those of the control sample. Furthermore, it becomes evident that increasing the recycled PET content as a bitumen replacement from 10 % to 15 % (mixes 10P-0CA and 15P-0CA, respectively) does not improve the overall impact categories emission (Fig. 6a).

Likewise, a consistent improvement in environmental performance is evident in cases where natural aggregates are substituted with carbonated aggregates (0P-15CA). This produces a significant impact, particularly reflected in the Climate Change and Marine Eutrophication results (Fig. 5). Due to the inherent characteristic of the carbonated aggregate of absorbing carbon, the effect of counteracting the emissions associated with aggregate quarrying and production can be seen in Fig. 7, where effectively, there is no emission associated with that material. On the contrary, when assessed against the replacement of bitumen or plastics, the environmental impact of CA's exclusive addition to the asphalt mix is relatively less significant. This neutral effect of CA on the mixes can be seen in the Freshwater Eutrophication and Marine Eutrophication associated emissions (Fig. 8, Fig. 9). Yet, it continues exhibiting positive environmental advantages compared to the control sample.

As can be anticipated, combining recycled plastics and carbonated aggregate has a significantly positive impact on gas emissions, with 10P-27CA and 10P-50CA being the most noteworthy, with 40 and 60 % reduction in Climate change emissions. The combined effect of more than halving the mixing process required energy by using WM, replacing the environmentally expensive bitumen with plastic, and eliminating the burdens associated with natural aggregate use with CA replacement can be seen in Fig. 7. In contrast, however, carbonated aggregate does not have the same significantly positive impact on Freshwater Eutrophication (Fig. 8), and it displays a counter effect when considering Marine eutrophication-associated emissions (Fig. 9). Nevertheless, compared to the control sample, the SRPS mixes' environmental performance remains positive, displaying an approximate 25 % decrease in impact due to the beneficial combination of recycled plastic and warm mixing techniques. (Fig. 6b).

These findings unequivocally demonstrate the profound positive impact on Climate Change and the other impact categories, from adopting SRPS mixes. These significant benefits reinforce these mixtures' environmental merit, highlighting their worthiness as sustainable alternatives.

According to the findings of [39], recycled plastic-enhanced asphalt provides environmental benefits relative to the traditional Hot Mix Asphalt. This includes 12.5 % savings in pavement thickness or extension of maintenance cycles by 7 %. The findings in Fig. 5, Fig. 6, Fig. 7, Fig. 8, Fig. 9 corroborate the findings of [39] and extend the findings by providing quantifiable evidence of the environmental benefits of plastic-polymer-modified asphalt pavements.

### 3.2 Comparison of the environmental impacts of PET closed and open loop recycling

**Fig. 10:**
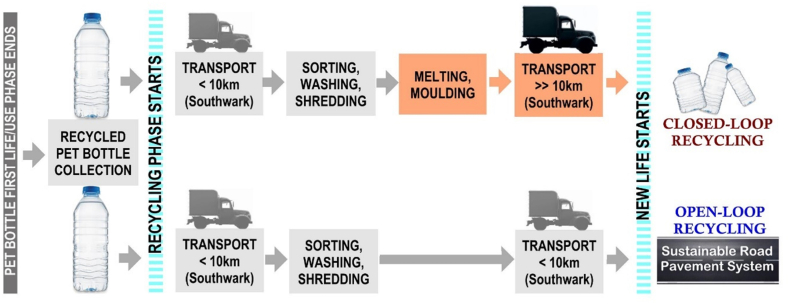
– Processes for PET open-loop and close-loop recycling.

This study and the results of the LCA displayed in the previous sections clearly show that using recycled PET as a substitute for bitumen in mixes used in road asphalt has an excellent environmental effect. However, the advantages of using recycled plastics in the mixes extend to a more holistic view that plastic waste can be reduced by adopting the innovative SRPS proposed in this study. Moreover, incorporating secondary materials like recycled plastics into asphalt mixes eliminates two environmentally expensive processes – the melting and moulding activities required when recycling plastic in a closed-loop scenario (Fig. 10). Furthermore, this study proposes implementing a plastic recycling and asphalt production infrastructure that uses recycled PET and produces pavement mixes locally to where the secondary materials are collected, and the roads are resurfaced, i.e., a London Borough (Southwark in this study). The added advantage of implementing the proposed “borough's local SRPS open-loop cycle infrastructure” is the associated transport distances and emission reductions (Fig. 10).

There is an urgent demand from the international research community to influence policymaking and current circular economy frameworks to implement more efficient plastic waste management scenarios [40]. The SRPS open-loop recycling can beneficially influence the UK's circular economy guidelines by reevaluating existing policies and adopting these alternative proposed strategies.

**Fig. 11:**
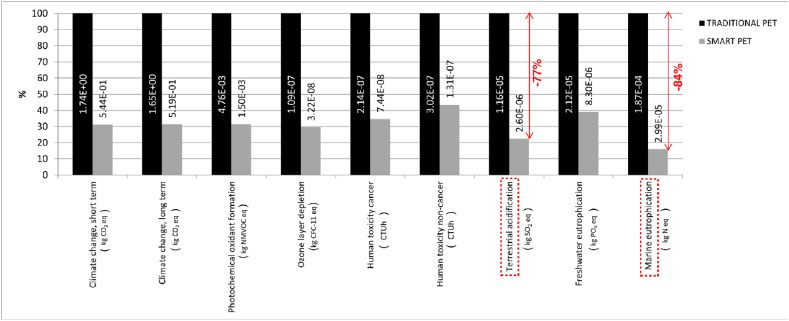
– Comparison of the environmental impact of traditional PET recycling and SRPS recycling.

**Fig. 12:**
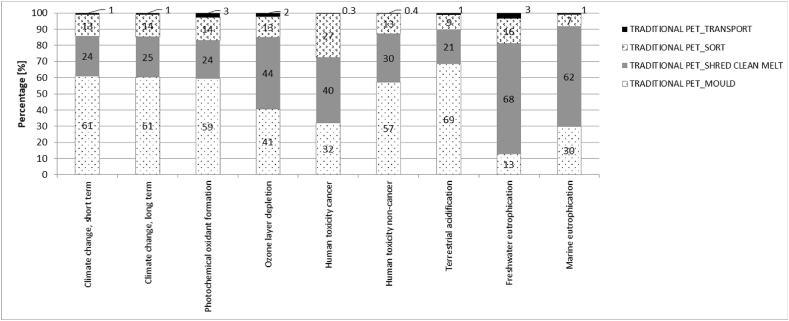
– LCA results for traditional PET closed-loop recycling and bottle production.

The associated benefits of favouring the SRPS over traditional plastic recycling are also displayed and summarised in Fig. 11, Fig. 12. In these figures, the environmental effect of traditional closed-loop recycling (producing new PET bottles), is compared to that of the open loop proposed in this study. The analysis results are significant and display a noteworthy 60–70 % reduction in emission across all impact categories.

The worthy environmental emissions improvement from the SRPS open cycle recycling decision can be attributed to eliminating the need to melt and mould the recycled material for new bottle production. For example, for traditional PET, 61 % of Climate Change and 69 % of Terrestrial acidifications emissions, are due to moulding processes associated with new bottled production (Fig. 12). The absence of this process for open-loop recycling processes is responsible for curbing a substantial portion, ranging from 24 % to 40 % of the overall emissions associated with traditional plastic bottle manufacturing across various impact categories. Notably, for Terrestrial acidification and Marine Eutrophication, this reduction exceeds 60 %, as depicted in Fig. 11.

## 4 Uncertainty analysis

**Fig. 13:**
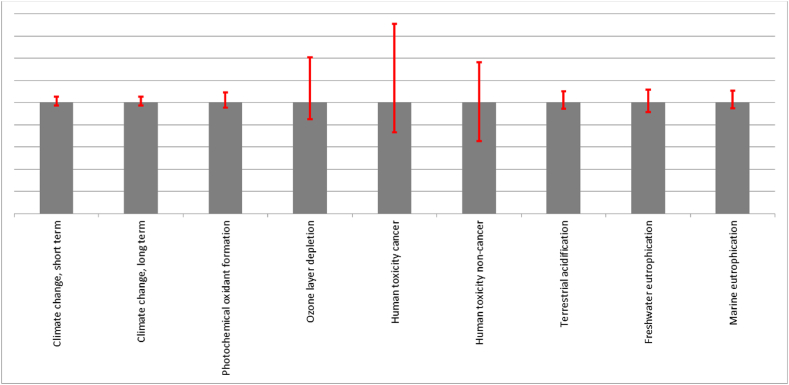
Uncertainty analysis of 10P-27CA mix using IMPACT World + Midpoint v1.01 with a confidence interval of 95 %.

In relation to the Life Cycle Assessment (LCA) result reliability, it is broadly recognised that uncertainty analysis should be conducted in an LCA study [20,21,34,41]. Consequently, a simulation of the inventory data is conducted using a Monte Carlo approach to ensure the results’ reliability for the best performing of the mixes analysed, SRPS 10P-27CA. The analysis identifies the most sensitive input parameters contributing to the output uncertainty, these are highlighted in red in Fig. 13 and show a potentially unreliable LCA result for Human toxicity and Ozone layer depletion.

**Fig. 14:**
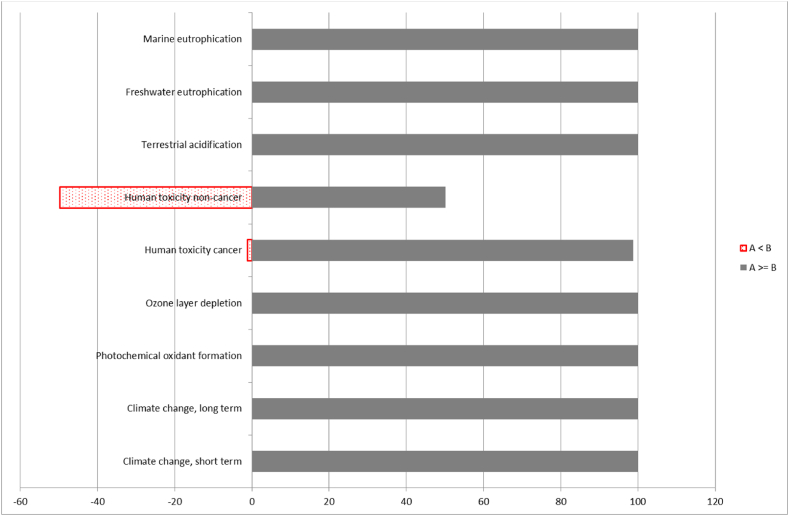
Comparative Montecarlo analysis of Control Sample CS and SRPS 10P-27CA mix; Confidence interval 99 %.

In Fig. 14, the findings validate the outcomes depicted in Fig. 13, where an uncertainty analysis comparison on the impact category level between RPS mix CS and SRPS mix 10P-27CA, respectively, the control sample and one of the best performing mixes in this study, are displayed. These results are obtained after 1000 iterations and random sampling for the input parameters from normal distributions. Each bar represents an impact category, and the bars on the left-hand side of Fig. 14 show the number of times the scenario without recycling had a lower load than the life cycle with recycling. Therefore, recycling would be better for all impact categories except for the remaining uncertainty on the Human toxicity and Ozone layer depletion highlighted by the red bars (Fig. 14).

The uncertainty analysis results, however, even though they must be considered, do not affect the overall positive outcome of the SRPS mixes environmental performance as Human toxicity and Ozone layer depletion were only displaying minimal positive effects in the LCA (Fig. 5). Furthermore, the other impact categories showing significant positive environmental performance show good reliability, confirmed by the Montecarlo analysis.

## 5 Research Outlook

By comparing the traditional Road Pavement Systems (RPS) environmental emissions with those of the SRPS, it is evident that the latter can significantly reduce energy consumption and emissions associated with asphalt production. Furthermore, by combining the use of plastic bottle (PET) waste as a binder in the mix with carbonated aggregate the demand for natural resources can be also reduced However, the importance of evaluating the environmental impact of both emerging and existing technologies beyond their greenhouse gasses emissions has been emphasised by the intergovernmental organisation [42], and international institutions [[43], [44], [45], [46], [47]]. Therefore, the scalability of the carbonated aggregate production methods to the UK and in the future, globally, must be further understood and remain outside the scope of this paper. In future studies, the cost-effectiveness, and social impact of producing carbonated aggregate on a larger scale and using open-loop recycled PET for SRPS production, must be assessed.

The environmental impacts associated with the recycled materials have been evaluated using a cut-off allocation method. However, alternative approaches should be evaluated similarly to studies already conducted for recycled concrete by Marinkovi et al. [48]. For example, by comparing results from cut-off to those of economic allocation, the benefits of increasing or reducing plastic recycling on a larger scale can be better evaluated and provide a broader context.

The system boundary and location and sourcing of the plastic material considered is adequate for these preliminary considerations into the SRPS technology, nonetheless, further studies should extend the analysis to cradle-to-cradle and work with local authorities to implement an efficient circular economy framework for plastic recycling. Data should be gathered from recycling centres to understand the scale of plastic recycling and the extent of local London boroughs’ road resurfacing annually.

The Montecarlo analysis findings of this study validate the significant reductions in emissions associated with the best-performing mix and related to Climate Change, Marine Eutrophication, and Freshwater Eutrophication. Nonetheless, further sensitivity analysis of all the mixes should be conducted in future studies to evaluate the differences across all mixes [49,50].

## 6 Conclusions

This research paper examines the results of a comparative LCA conducted on eight distinct pavement systems. These proposed asphalt mixes incorporate varying amounts of recycled PET and CA to partially substitute bitumen and aggregate. The need for more sustainable asphalt production drives the concept of sustainable road pavement systems (SRPS) proposed in this study.

The study aimed to conduct the LCA of the SRPS mixes and compare their environmental performance with a control sample representing the traditional asphalt mixes. The analysis considered the environmental burden of sourcing virgin materials, processing them for their final use in an asphalt mix and the energies required to mix the constituents. Concerning secondary materials, such as PET recycled plastic and carbonated aggregate, the assumptions made in the study were that of cut-off allocation, excluding the environmental burden associated with the product's first life. The analysis presented in this paper was carried out using Simapro software in combination with an IMPACT World + Midpoint method, and data was sourced using the Ecoinvent database and, when not available, literature and survey data as applicable.

Using a cradle-to-gate approach and mass allocation for the output products from virgin materials, the LCA results display promising environmental benefits. Using a circular economy approach in material sourcing through adopting carbonated aggregate and recycled PET, along with warm mixing techniques to decrease the required asphalt mixing energy, results in significant emission reductions. These reductions range from 40 % to 60 % for Climate Change, up to 30 % for Marine eutrophication, and up to 20 % for Freshwater eutrophication, as indicated by this study.

Moreover, the robustness of the LCA result outcomes underwent additional validation by implementing a Montecarlo analysis. This additional reliability analysis instills confidence in the credibility of the presented results, especially for impact categories exhibiting notable positive environmental effects, such as Climate Change, Freshwater eutrophication, and Marine Eutrophication.

The findings from this study have some limitations. The LCA study focused on when the asphalt road pavement mixes are produced to when they are laid. A further LCA analysis is recommended from when the asphalt road pavement mixes are laid to their end of life when they will be recycled.

## Data availability statement

Data will be made available on request.

## Additional information

No additional information is available for this paper.

## CRediT authorship contribution statement

**Ottavia Rispoli:** Methodology, Software, Validation, Writing – original draft. **Oluwatoyin Opeyemi Ajibade:** Conceptualization, Funding acquisition, Investigation, Methodology, Project administration, Supervision, Validation, Writing – review & editing.

## Declaration of competing interest

The authors declare that they have no known competing financial interests or personal relationships that could have appeared to influence the work reported in this paper.
